# Determinants of precancerous cervical lesion among HIV infected women on ART in Woldia comprehensive specialized hospital NorthEast Ethiopia

**DOI:** 10.1186/s12905-023-02580-0

**Published:** 2023-08-29

**Authors:** Tazeb Melkie Dessie, Abebe Tarekegn Kassaw, Gedefaw Diress Alen

**Affiliations:** 1 Department of Public Health, Woldia Comprehensive Specialized Hospital, Woldia, Ethiopia; 2 Department of Pharmacy, College of Health Science, Woldia University, Po. Box: 400, Woldia, Ethiopia; 3 Department of Public Health, College of Medicine and Health Sciences, Debre Markos University, DebreMarkos, Ethiopia; 4 Center for Alcohol Policy Reseach, La Trobe University, Melbourne, Australia

**Keywords:** Precancerous cervical lesion, Human papillomavirus, Case-control, Risk factor, Woldia Hospital

## Abstract

**Background:**

Precancerous cervical lesion is a priority public health problem that jeopardizes the life of enormous women. previous studies in Ethiopia were more focused on knowledge, attitude, and practices of Cervical cancer screening. studies on the risk factors of pre-cancerous cervical lesions among the risk population (HIV infected) relative to the general population were limited. This study aimed to identify the determinants of precancerous cervical lesions among HIV Infected Women in Woldia Comprehensive Specialized Hospital in Northeast Ethiopia, 2022.

**Methods:**

Hospital-based unmatched case-control study was conducted in Woldia Comprehensive Specialized Hospital among HIV-infected women from June to August 2022. Data were collected from 104 cases and 208 controls using an interviewer-administered questionnaire and clinical data from the patient chart using Electronic Medical Record _ Anti-Retroviral Therapy Smart care database checklist. The binary logistic regression model was used to identify the determinants of the precancerous cervical lesion. An odds ratio with a 95% Confidence interval was used to measure the association and p-value < 0.05 were considered significant.

**Results:**

Women who have two or more lifetime sexual partners (AOR = 3.21,95% CI: 1.71–6.04), history of sexually transmitted infection (AOR = 4.97, 95% CI: 2.78–8.78), early age at first sexual intercourse (< 18 years) (AOR = 4.35,95% CI: 2.48–7.67) and baseline CD4 count < 200 cells/mm3 (AOR = 1.89, 95% CI: 1–3.57) had a higher odd of developing a precancerous cervical lesion.

**Conclusion:**

This study confirms that having a history of sexually transmitted infection, two or more lifetime sexual Partners, the initiation of sexual intercourse before the age of 18 years, and Baseline CD4 count < 200 cells/mm3 were determinants for precancerous cervical lesions. So it should be focused on prevention through early detection and treatment of sexually transmitted infections.

## Background

Cervical cancer (CC) is the fourth most common cancer among women following breast cancer, colorectal cancer, and lung cancer in the globe [1]. According to previous reports, CC is the second-top deadly cancer among Ethiopian women [2, 3]. In 2016 alone, an estimated 718,500 people were living with HIV(PLHIV ), women accounted for 60% of all HIV infections (about 433,763 women were living with HIV) [4]. Similarly, recent reports have indicated that CC is the leading cause of cancer-related death in low and middle-income countries [1, 5]. The poor socio-economic status, less accessible and underdeveloped healthcare system, and the high HIV/AIDS burden in sub-Saharan Africa have potentially increased the incidence of CC in the region [6]. Due to this increment in CC incidence, women in this region are more affected with greater morbidity and mortality rates relative to other regions of the world [7].

According to World Health Organization (WHO) estimation, CC is expected to kill over 443,000 women by 2030, and the majority (more than 98% of deaths) are believed to occur in developing countries (mainly in SSA) [6]. The current recommended CC screening method in resource-limited settings like SSA is a visual inspection of the cervix using acetic acid [6, 8]. Besides, using rapid, easy-to-use, and low-cost molecular tests; such as the OncoE6 test directly detects the elevated levels of the E6 oncoprotein of Human Papilloma Virus (HPV) types 16 and 18 can be used as a preferred screening test over previous screening method [9].

Cervical cancer is a priority public health problem that causes high morbidity and mortality among women in the developed and developing world [10]. Sub-Saharan, particularly Eastern African countries carry the highest burden which accounts for about (23.3%) of morbidity and (16.54%) of mortality [10–12]. The overall burden of CC is projected to continue to rise [13]. It is the third most common cancer among women worldwide ranging from 2.4 to 25.2% while secondly ranked leading cancer in Africa, which accounts for 25.2% [14]. In Ethiopia, because of the silent nature of the disease until it is quite advanced and the presence of large gaps in women’s level of awareness of the responsible factors, women are tragically dying of cervical cancer [12, 15, 16]. Every year 6294 women are diagnosed with a *precancerous cervical lesion* (PCL); of them, Around 4884 women died of it [12, 17]. The frequency of PCL was 6.7–22.1% and 15.9% across the country and Amhara region respectively, with considerably higher prevalence among HIV-positive women [18]. To address this serious public health problem, Ethiopia adopted Sustainable Development Goal (SDG), and World Health Organization (WHO) strategies and designed the National Cancer Control Plan (NCCP) [19].

WHO and the Ethiopian Federal Ministry of Health (FMOH) recommends the early identification of premalignant lesions through visual inspection with acetic acid [1, 20]. And apply the “screen and treat” approach to preventing invasive cervical cancer in high-risk women [21–23]. Yet, cervical cancer continues to be the leading female malignancy because of large gaps in responsible risk factors for PCL [12]. VIA is the preferred screening approach for cervical pre-invasive lesions since it is an evidence-based affordable alternative in low-resource settings including Ethiopia [11, 23, 24]. Factors accompanying PCL are suggested as socio-demographic, reproductive lifestyle, and sexual behavior-related factors. However, factors associated with premalignant cervical lesions remain poorly documented [25, 26].

Despite, recognizing risk factors with PCL having a supreme imperative for designing more targeted screening programs to reduce severe morbidity and mortality, most epidemiologic research has been done on invasive cervical cancer [22, 27, 28]. The cause of PCL has been the subject of several studies in the last three decades and cervical cancer incidence has declined in developed countries. However, the growing pattern of cervical cancer cases in Africa including Ethiopia is substantially increasing [29–31].

Awareness creation on the availability of screening and treatment services is also necessary. lack of knowledge about the disease and risk factors, beliefs about the disease, poor access to preventive services, and the current health service system can affect the decision to be screened for cervical cancer [32]. because of the silent nature of the disease until it is quite advanced and the presence of large gaps in women’s level of awareness of the responsible factors, women are tragically dying of cervical cancer. It is one of the preventable leading causes of death among women. Thus, the identification of risk factors associated with precancerous lesions is essential to take primary preventive measures and tackle the progress to invasive cancer [12].

women are at increased risk of acquiring human papillomavirus infection, pre-cancerous cervical lesion, and even having an invasive CC compared with uninfected women [33, 34]. Therefore, this study aimed to identify factors associated with precancerous Cervical Lesions and it helps program designers in designing more targeted programs for cervical cancer prevention and control.

## Methods and materials

### Study area and setting

The study was conducted at Woldia Comprehensive Specialized Hospital (WCSH), which is located in Woldia town 521 km away in the northeast of Addis Ababa, the capital city of Ethiopia, and 360 Km from the Regional capital city, Bahir Dar. The WCSH provides Emergency, outpatient, and inpatient services for more than two million people living in its catchment. Among the different services, the one is ART service, which starts on September 1998 E.C. Currently 4000 clients have on follow-up, of which 89 were pediatric and 3911 were adult clients, 1570 are male and 2341 are female. Among 2341 female clients 1400 clients who have eligible for cervical cancer screening services. The hospital provides cervical cancer prevention services and treatments available to treat CIN: cryotherapy, thermal ablation, large loop excision of the transformation zone, and punch biopsy for a suspicious lesion by VIA screening.

### Study design and period

An institutional-based unmatched case-control study was conducted among HIV-positive women attending the adult ART clinic at Woldia comprehensives specialized hospital from June 16 to August 15, 2022, Northeast Ethiopia.

### Population

#### Source population

All adult Female PLHIV clients aged between 15 and 49 years old attending Woldia Comprehensive Specialized Hospital.

#### Study population

The study participants were adult Female clients with age between 15 and 49 and receiving care or actively taking ARV drugs at Woldia Comprehensive Specialized Hospital ART clinic during the study period.

#### Inclusion criteria

All HIV-infected women aged 15-49-year-old, who will be screened for cervical cancer at ART clinic using VIA in Woldia Comprehensive Specialized hospitals during the data collection period were included in the study.

#### Exclusion criteria

Clients who were absent during the data collection time (Dead, transfer out Lost to Follow-up and Stop), critically ill, and unable to communicate were excluded from the study.

#### Sample size determination

The Sample size for the study was calculated by using the double Population proportion formula with Epi info version 7.2.5 software, by considering the following assumptions; 95% level of significance, 80% power, the ratio of control to case 2:1, the proportion of exposed to controls 66.7%, Odds ratio 2.1, percent of cases with exposure 33.3% from the previous similar study was done in five health facilities in south Ethiopia [35]. thus, the largest sample size obtained for this study was 284, by considering a 10% non-response rate the final minimum adequate sample size was estimated to be 312 individuals (104 cases and 208 controls).

**Table:** 

Variables	Level of significance	Power	OR	Ratio	Sample size
Marital Status	95%	80%	3.3	1:2	119
History of STI	95%	80%	2.1	1:2	284
Age	95%	80%	2.5	1:2	192
Lifetime sexual partners	95%	80%	4.7	1:2	78

### Operational definitions

#### Precancerous cervical lesion positive

when an acetowhite lesion with well-defined margins is observed within the vicinity of the transformation zone, and/or if the whole cervix turned white by VIA screening (VIA positive for precancerous cervical lesion) [29].

#### Precancerous cervical lesion negative

when there is no acetowhite lesion on the surface of the cervix by VIA screening (VIA negative for precancerous cervical lesion) [29].

#### VIA positive

the presence of raised and thickened white plaques and/or acetowhite epithelium, near the squamocolumnar junction (SCJ) of the cervix during VIA screening [29].

#### VIA negative

the presence of smooth, pink, uniform, and featureless cervix during VIA screening [29].

#### Case

women those positive for precancerous cervical lesion using VIA screening (VIA- positive for precancerous cervical lesion).

#### Control

women those negative for precancerous cervical lesions using VIA screening (VIA negative for precancerous cervical lesion).

### Data collection tool and procedure

A structured interviewer questionnaire was constructed by reviewing similar articles. The questionnaire was prepared in English, then translated into Amharic, and then into English to maintain consistency. The questionnaire included important variables such as Respondent sociodemographic information, reproductive health-related variables, and other hygiene-related factors. Three BSC midwife data collectors and one medical oncology supervisor were assigned. Data collectors had relatively similar professional experience in screening services. They also had experience collecting data on the same topic from other areas of the healthcare facility. The cervical lesion was screened by trained health professionals. The training was conducted for two days in the form of interviews and filling out questionnaires. According to the World Health Organization (WHO) guidelines for screening and treatment of precancerous lesions of cervical cancer prevention [20], the result can be interpreted as positive when an acetowhite lesion with well-defined margins is observed within the vicinity of the transformation zone, on the other hand, if the whole cervix turned white (visual inspection with acetic acid—positive). the result would be negative when there is no acetowhite lesion (visual inspection with acetic acid—negative); or suspicious for cancer when there is a visible ulcerative cauliflower-like ulcer, oozing, and bleeding on touch. Women with findings of “suspicious” were not included in the study.

### Data analysis

Data were entered, cleaned, checked, edited, and coded into Epi Data version 3.1 and analyzed using SPSS version 25 statistical software. Categorical variables were summarized as frequencies and percentages. Descriptive statistics were computed and reported by using frequency, mean, standard deviation, and percentage. *A binary logistic regression model was used to identify the determinants of precancerous cervical lesions. An odds ratio with a 95% Confidence interval was used to measure the association*. The model fitness was checked by using the Hosmer-Lemeshow goodness of fittest. Data were interpreted by using an Odds ratio with a 95% confidence interval and a P-value < 0.05 were taken as statistically significant in the final multivariable logistic regression model.

## Result

### Socio-demographic characteristics

A total of 312 women who were on ART were included with a response rate of 100%. The mean age of the cases and the controls was 37 years (SD: ±8 years) and 38 years (SD: ±8 years) respectively. One hundred forty-two (45.5%) and more than half of 175 (56.1%) of the study participants were elementary in educational status and Married Respectively. One hundred fifty-two (48.7%) and 190 (60.9%) study participants were Orthodox followers ‘and urban residents, respectively (Table 1).

**Table 1:** Socio-demographic characteristics of study participants among HIV-positive women in Woldia Comprehensive Specialized hospital northeast Ethiopia, 2022

Variables	Frequency	Present
**Age in Year**		
15–29	41	13.1
30–39	182	58.3
40–49	89	28.5
**Educational status**		
Unable to read and write	39	12.5
Elementary(1-8grade)	142	45.5
Secondary and above (9–12 grade)	78	25
Certificate and above	53	17
**Marital Status**		
Single	19	6.1
Married	175	56.1
Widowed	45	14.4
Divorced	73	23.4
**Occupation**		
Housewife	53	17
Merchant	51	16.3
Daily labor	58	18.6
Governmental employee	74	23.7
Private /NGO employee	76	24.4
**Religion**		
Orthodox	152	48.7
Muslim	118	37.8
Protestant	26	8.3
Catholic	16	5.1
**Residence**		
Rural	122	39.1
Urban	190	60.9

### Reproductive health characteristics

The proportion of contraceptive use was 19.9% and 37.2% among the cases and controls respectively. among 89 (28.5%) cases and 174 (37.2%) of the controls have seen their first menarche at less than 15 years and also 22.1%) of the cases and 51% of the controls had regular Menstrual bleeding patterns. Age at first marriage 47 (15.1%) of the cases and 77 (24.7%) of the controls between 20 and 24 years. Among 33 (10.6%) of the cases and 83 (26.6%) of the controls had 3–4 children (Table 2).

**Table 2:** Reproductive health characteristics of study participants among HIV-positive women in Woldia Comprehensive Specialized Hospital northeast Ethiopia, 2022

Variables	Case(n = 104)	Control (n = 208)	Total (N = 312)
**Use of Contraceptive**	**n (%)**	**n (%)**	**n (%)**
Yes	61 (19.9)	116 (37.2)	178 (57.1)
No	42(13.5%)	92 (29.5)	195(42.9%)
**Age at menarche**			
< 15 years	89 (28.5)	174 (55.8)	263 (84.3)
15–17 years	15 (4.8)	34 (10.9)	49 (15.7)
**Menstrual bleeding pattern**			
Regular	69 (22.1)	159 (51)	228 (73.1)
Irregular	35 (11.2)	49 (15.7)	51 (26.9)
**History of postcoital bleeding**			
Yes	8 (2.6)	17 (5.4)	25 (8)
No	96 (30.8)	191 (61.2)	287 (92)
**Age at first marriage**			
10–14	6(1.9)	20(6.4)	26(8.3)
15–19	24(7.7)	48(15.4	72(23.1)
20–24	47(15.1)	77(24.7)	124(39.7)
25–29	27(8.7)	63(20.2)	90(28.8)
**Parity (delivery experience)**			
No	30 (9.6)	72 (23.1)	102 (32.7)
1–2	34 (10.9)	41 (13.1)	75 (24)
3–4	33 (10.6)	83 (26.6)	116 (37.2)
>=5	7 (2.2)	12 (3.8)	19 (6.1)
**History of Abortion**			
Yes	14 (4.5)	20 (6.4)	34 (10.9)
No	90 (28.8)	188 (60.3)	278 (89.1)

### Lifestyle and sexual behavior

In the majority of the cases 24.4% and controls 47.8% had sometimes used a condom in their lifetime. Fifteen (4.8%) of the cases and 15(4.8%) of the controls had a Family History of Cervical Cancer The magnitude of having lifetime multiple (two or more) sexual partners among cases and controls was 66 (21.2%) and 171 (54.8%) respectively. Besides, 51 (16.3%) of the cases and 170(54.5%) of the controls had a history of sexually transmitted infections (STI). In seventy (22.4%) of the case and 76 (24.4%) of the controls, the respondents started their first sex at the age < 18 but other respondents started their first sex at the age greater than 18 years (10.9%) and (42.3% in cases and controls respectively (Table 3).

**Table 3:** Lifestyle and sexual characteristics of study participants among HIV-positive women in Woldia Comprehensive Specialized hospital northeast Ethiopia, 2022

Variables	Case (n = 104)	Control (n = 208)	Total (N = 312)		
**Condom used during sexual intercourse**	**n (%)**	**n (%)**	**n (%)**		
Always	20 (6.4)	37 (11.9)	57 (18.3)		
Sometimes	76 (24.4)	149 (47.8)	225 (72.1)		
Never use	8 (2.6)	22 (7.1)	30 (9.6)		
**Ever smoked**					
Yes	9(2.9)	11(3.5)	20(6.4)		
No	95(30.4)	197(63.1)	292(93.6)		
**Ever drunk alcohol**					
Yes	8(2.6)	10(3.2)	21(5.8)		
No	96(30.8)	198(63.5)	291(94.3)		
**Family History of Cervical Cancer**					
Yes	15(4.8)	15(4.8)	30(9.6)		
No	89(28.5)	193(61.9)	282(90.4)		
**Number of Lifetime Sexual Partners**					
Two or More	66(21.2)	171(54.8)	237(76)		
No or one	38(12.2)	37(11.9)	75(24)		
**Ever had a History of STI**					
No	53(17)	38(12.2)	91(29.2)		
Yes	51(16.3)	170(54.5)	221(70.8)		
**Partner History of STI**					
No	42(13.5)	89(28.5)	131(42)		
Yes	61(19.9)	119(38.1)	181(58)		
**Do your partners have another partner**					
Yes	22(7.1)	33(10.6)	55(17.6)		
No	52(16.7)	122(39.1)	174(55.8)		
Unknown	30(9.6)	53(17)	83(26.6)		
**Age at first sexual intercourse**					
< 18	70(22.4)	76(24.4)	146(46.8)		
>=18	34(10.9)	132(42.3)	166(53.2)		
**BMI**					
Underweight	9(2.9)	20(6.4)	29(9.3)		
Normal	73(23.4)	134(42.9)	207(66.3)		
Over Weight	22(7.1)	54(17.3)	76(24.4)		

### Clinical and health-related factors

**Fig. 1:**
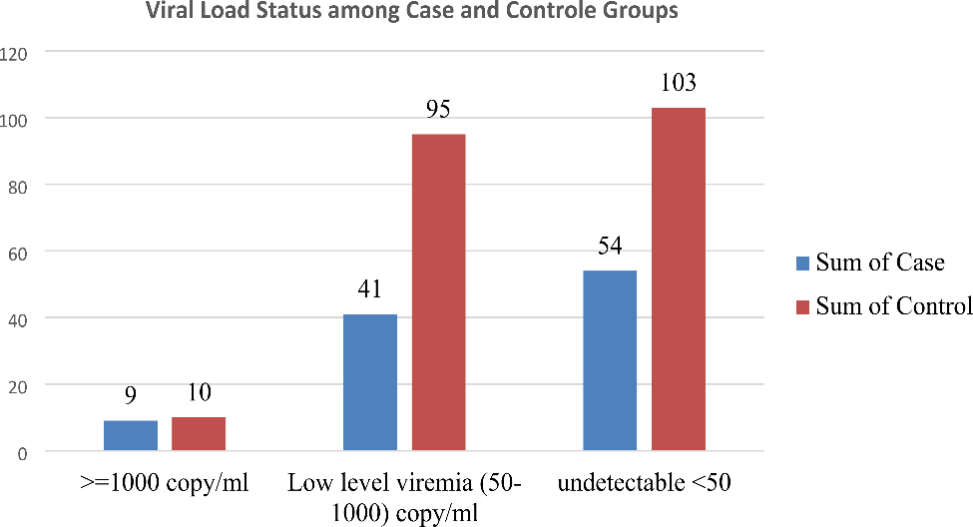
Viral Load Status with precancerous cervical lesion among HIV-positive women in Woldia Comprehensive Specialized hospital northeast Ethiopia, 2022

The majority of the duration of Antiretroviral Therapy of the respondents of the cases 49(15.7%) and controls 116(37.2%) were 12–36 Months. Among 65(20.8%) of the case and 112(35.9%) of the control WHO Stage T1. 28(9%) of the case and 38(12.2%) of the control were baseline CD4 count greater than 200 cells/mm3. The current viral load status of the respondents 54(17.3%) of the cases and 103 (33%) of the controls were Undetectable (< 50) copies/ml whereas 41(95%) and 95(30.4%) shows Low-level viremia (50-1000) copy’s/ml in cases and controls respectively (Figure-1). the majority of the respondents 71(22.8%) of the cases and 152(48.7%) of the controls were in the first-line Regimen (Table 4).

**Table 4:** Healthcare factors associated with precancerous cervical lesion among HIV-positive women in Woldia Comprehensive Specialized hospital northeast Ethiopia, 2022

	Case(n = 104)	Control (n = 208)	Total (N = 312)
**Duration of Antiretroviral Therapy**	**n (%)**	**n (%)**	**n (%)**
< 12 Month	4(13)	17(5.4)	21(6.7)
12–36 Months	49(15.7)	116(37.2)	165(52.9)
> 36 Months	51(16.3)	75(24)	126(40.4)
**WHO Stage**			
T 1	65(20.8)	112(35.9)	177(56.7)
T 2	21(6.7)	4514.4)	66(21.2)
T 3	12(3.8)	38(12.2)	50(16)
T 4	6(1.9)	13(4.2)	19(6.1)
**Baseline CD4 cell count (cells/mm3)**			
>=200	28(9)	38(12.2)	65(21.2)
< 200	76(24.4)	170(54.5)	246(78.8)
**HAART Adherence**			
Good	88(28.2)	168(53.8)	256(82.1)
Fair	9(2.9)	28(9)	37(11.9)
Poor	7(2.2)	12(3.8)	19(6.1)
**Regimen Line**			
First line Regimen	71(22.8)	152(48.7)	223(71.5)
Second line Regimen	33(10.6)	56(17.9)	89(28.5)
**Chronic corticosteroid use**			
Yes	7(2.2)	15(4.8)	22(7.1)
No	97(31.1)	193(61.9)	290(92.9)

### Factors associated with precancerous cervical lesion

In the bivariable logistic regression analysis, the precancerous cervical lesion was associated with Age in Year, educational status, family History of Cervical Cancer, number of Sexual Partner, every History of STI, age at first sexual intercourse, Duration of Antiretroviral Therapy, WHO Stage and Baseline CD4 count < 200 cells/mm3, at a p-value of < 0.2. However, in the multivariate analysis which was done to adjust for potentially confounding variables, four explanatory variables (the number of Sexual Partner, ever History of STI, Age at first sexual intercourse, and baseline CD4 count < 200 cells/mm3 were significantly associated with a precancerous cervical lesion at p-value < 0.05.

The odds of having a precancerous cervical lesion were 3.21 times higher among women who had two or more lifetime sexual partners as compared to those who had less than two-lifetime sexual partners (AOR = 3.21,95% CI (1.71–6.04)).

The odds of developing precancerous cervical lesions among women who had a history of STI was 4.97 times higher as compared to those women who had no history of STI (AOR = 4.97,95% CI (2.78–8.78)).

The odds of having a precancerous cervical lesion are 4.35 times higher among women who had early initiated sexual intercourse before the age of 18 as compared to those who had initiated sexual intercourse at the age of 18 years and late (AOR = 4.35, 95% CI (2.48, 7.67)).

The odds of developing precancerous cervical lesion among women who had baseline CD4 count < 200 cells/mm3 was 1.89 times the odds of those women who had baseline CD4 count > = 200 cells/mm3 (AOR = 1.89, 95% CI (1-3.57)) (Table 5).

**Table 5:** Bivariable and Multivariable analysis of logistic Regression factors associated with precancerous cervical lesion among HIV-positive women in Woldia Comprehensive Specialized Hospital northeast Ethiopia, 2022 (n = 312)

Variables	Case n (%)	Control n (%)	COR (95%CI)	AOR (95%CI)	P values
Age in Year					
15–29	17(5.4)	24(7.7)	1	1	
30–39	54(17.3)	128(41)	1.679(0.84–3.37*	1.49(0.67–3.3)	0.33
40–49	33(10.6)	56(17.9)	1.2(0.56–2.56)	0.77(0.32–1.83)	0.55
Educational status					
Unable to read and write	17(5.4)	22(7.1)	0.56(0.24–1.33) *	0.71 (0.23–2.15)	0.47
Elementary(1-8grade)	44(14.2)	98(31.4)	0.96(0.485–1.91)	1.28(0.54–3.06)	0.57
Secondary and above (9–12 grade)	27(8.7)	51(16.3)	0.82(0.386–1.73)	0.67(0.26–1.72)	0.406
Certificate and above	16(5.1)	37(11.9)	1	1	
Family History of Cervical Cancer					
Yes	15(4.8)	15(4.8)	0.46(0.22–0.99) *	0.47(0.18–1.23)	0.13
No	89(28.5)	193(61.9)	1	1	
Number of Sexual Partners					
Two or More	66(21.2)	171(54.8)	0.51(0.23–1.12) *	**3.21(1.71–6.04)**	0.00**
No or one	38(12.2)	37(11.9)	1	1	
Ever had a History of STI					
No	53(17)	38(12.2)	1	1	
Yes	51(16.3)	170(54.5)	4.65(2.67–7.83)	**4.97(2.78–8.78)**	0.00**
Age at first sexual intercourse					
>=18	70(22.4)	76(24.4)	1	1	
< 18	34(10.9)	132(42.3)	3.78(2.2–5.88) *	4.35(2.48–7.67) *	0.00**
Duration of Antiretroviral Therapy					
< 12 Month	4(13)	17(5.4)	1	1	
12–36 Months	49(15.7)	116(37.2)	0.806(0.3–2.2)	0.68(0.22–2.1)	0.503
> 36 Months	51(16.3)	75(24)	0.49(0.18–1.33) *	0.4(0.13–1.23)	0.11
WHO Stage					
T 1	65(20.8)	112(35.9)	1	1	
T 2	21(6.7)	4514.4)	1.24(0.68–2.27)	0.96(0.45–2.04)	0.92
T 3	12(3.8)	38(12.2)	1.84(0.89–3.76)	1.79(0.75–4.29)	0.19
T 4	6(1.9)	13(4.2)	1.84(0.897–3.766) *	3.15(0.96–10.3)	0.05
Baseline CD4 cell count (cells/mm3)					
>=200	28(9)	38(12.2)	1	1	
< 200	76(24.4)	170(54.5)	1.814(1.05–3.15) *	**1.89(1-3.57)**	0.047**

Where 1 = Reference, and

*= Shows the variable Significant at P Value < 0.2 in bivariable analysis

** = Shows the variable Significant at P Value < 0.05 in Multivariable analysis

## Discussion

This study identified the predictor of precancerous cervical lesions among HIV-infected women on ART that helps to identify the screening requirements and to take early preventive measures. In this study, different factors were identified which can be associated with the precancerous cervical lesion among HIV-infected women on ART.

Those Women who had multiple sexual partners, a history of sexually transmitted infections, had a history of early (before age 18 years) initiation of sexual intercourse, had Baseline CD4 count Less than 200/mm3 had a significant association with a precancerous cervical lesion.

The odds of having a precancerous cervical lesion were higher among women who had two or more lifetime sexual partners as compared to those who had less than two-lifetime sexual partners. The finding was comparable with a study conducted in Southern Ethiopia, Gahandi Memorial Hospital, and Addis Ababa that multiple sexual partners were a determinant factor for the development of precancerous cervical lesions [29, 35, 36]. And also, have been comparable with the study conducted in three referral Hospitals in Amhara Region, Bahrdar Town, and Gondar University Comprehensive Specialized Hospital [12, 37, 38]. This could be because women who have had 2 or more lifetime sexual partners are more likely to develop precancerous cervical lesions, as the number of sexual partners increases; they are more likely to contract the HPV infection, which is the cause of cervical cancer and invasive cervical cancer. However, other studies done in selected hospitals of West Wollega showed that having a History of multiple sexual partners was not associated with the risk of precancerous cervical lesion development [39]. The possible reason for the difference might be the difference in the awareness level of sexual partners and/or the awareness level of commercial sex workers on the transmission and prevention methods of STI.

Sexually Transmitted Infections (STIs) were another determinant factor for the development of precancerous cervical lesions among women in this study. The odds of developing pre-cancerous cervical lesions among women with a history of sexually transmitted infections were higher than among women who had no history of STIs. This finding was consistent with the studies conducted in Southern Ethiopia, Addis Ababa, three referral Hospitals in Amhara region, North Shoa, and Wollega which revealed that women who had a history of sexually transmitted infection were at higher risk of developing precancerous cervical lesions infection [12, 35, 39–41]. This could be due to the sexually transmitted nature of HPV infection. More than 90% of cervical cancers developed due to persistent infection of the human papillomavirus (HPV), which is most often transmitted through sexual intercourse [38]. This might be due to Long-term inflammation caused by sexually transmitted infections increasing the risk of precancerous cervical lesions.

Early initiation of sexual intercourse is associated with increased odds of acquiring pre-cancerous cervical lesions. The study found that women who initiated sexual intercourse before 18 years were more likely to develop pre-cancerous cervical lesions than women who started sexual intercourse after 18 years. The finding of this study was consistent with studies done in North shoa and Mari stope Ethiopia in Adama in which women who had a history of early initiation of first sexual intercourse before 15 were at higher risk to develop precancerous cervical lesion than those whose age is greater than 15 years age [24, 41].and also this study similar with the study conducted in Bahrdar Town And Gahandi Memorial Hospital early initiation of first sexual intercourse before the age of 18 were risk of developing precancerous cervical lesions compare with after the age 18 [36, 37]. This might be because at this age cervical tissue undergoes physiologic changes, the transformation zone on the ectocervix is enlarged, and exposure to HPV at such times may facilitate infection which may make this area more vulnerable to the development of dysplasia, a cervical squamous cancer. In addition, an earlier age of sexual practice implies a longer period of sexual activity which increases the chance of developing cervical cancer [36].

CD4 cell count was an additional factor associated with the precancerous cervical lesion. The odds of developing precancerous cervical lesions among women who had baseline CD4 cells less than 200/mm3 were more likely to have developed precancerous cervical lesions compared to those with baseline CD4 cells above 200/mm3. the finding was consistent with the study done in South Africa, sub-Saharan Africa, and three Referral Hospitals in Amhara Region [40, 42, 43]. Since the level of the CD4 count is a vital indicator of immune status, having a better immune status helps protect women from precancerous cervical lesions [42]. the other possible explanations might be due to the effect of immune function, HIV-induced immunosuppression leads to an inability to control the HPV expression, hence the persistence of HPV infection and the development of cervical lesions.

The limitation of this study was, Visual inspection with an acetic acid test result of postmenopausal women may not be reliable because the transformation zone of these women is often inside the cervical canal which may have an impact on the magnitude.in addition, few women had difficulty recalling their prior exposure to multiple sexual partners during the interview.

## Conclusion

This study revealed that having two or more sexual partners, having a history of a sexually transmitted infection, beginning a relationship before turning 18 years old, and having a baseline CD4 count below 200 cells/mm3 were indicators of precancerous cervical lesions. Precancerous cervical lesion reveals a significant public health problem among HIV-infected women unless special consideration is taken. Awareness creation on the availability of screening and treatment services is also necessary so that HIV-infected women could use the service. Hence, Health care providers, Hospital mentors, and partners need to conduct regular technical support to improve gaps in opportunistic infection prevention, Cervical cancer screening, diagnosis and treatment of Opportunistic infections, guidelines and tools with onsite supervision, and timely CD4 count-related gaps with appropriate feedback.

## Data Availability

The study materials and data are available from the corresponding author upon request.

## List of Acronyms and Abbreviations

ART: Anti-Retroviral Therapy
CIN: Cervical Intraepithelial Neoplasia
EMR: Electronic Medical Record
HMIS: Health Management Information System
HPV: Human Papilloma Virus
LEEP: loop electrosurgical excision procedure
NCD: Non-Communicable Disease
NCCP: National Cancer Control Plan
PEPFAR: United States President’s Emergency Plan for AIDS Relief
PCL: Precancerous Cervical Lesion
SDG: Sustainable Development Goal
STD: Sexual Transmitted Disease
VIA: Visual Inspection with Acetic acid
WHO: World Health Organization
